# Extracellular vesicles produced by the human gut commensal bacterium *Bacteroides thetaiotaomicron* elicit anti-inflammatory responses from innate immune cells

**DOI:** 10.3389/fmicb.2022.1050271

**Published:** 2022-11-10

**Authors:** Sonia Fonseca, Ana L. Carvalho, Ariadna Miquel-Clopés, Emily J. Jones, Rokas Juodeikis, Régis Stentz, Simon R. Carding

**Affiliations:** ^1^Gut Microbes and Health, Quadram Institute Bioscience, Norwich, United Kingdom; ^2^Department of Women’s and Children’s Health, Institute of Life Course and Medical Sciences, University of Liverpool, Liverpool, United Kingdom; ^3^Norwich Medical School, University of East Anglia, Norwich, United Kingdom

**Keywords:** extracellular vesicles, Bacteroides, anti-inflammatory response, innate immune tolerance, BMDM, THP-1 cells, TLR2, IL-10

## Abstract

Bacterial extracellular vesicles (BEVs) produced by gut commensal bacteria have been proposed to play an important role in maintaining host homeostasis *via* interactions with the immune system. Details of the mediators and pathways of BEV-immune cell interactions are however incomplete. In this study, we provide evidence for the anti-inflammatory and immunomodulatory properties of extracellular vesicles produced by the prominent human gut commensal bacterium *Bacteroides thetaiotaomicron* (Bt BEVs) and identify the molecular mechanisms underlying their interaction with innate immune cells. In mice treated with colitis-inducing dextran sodium sulfate (DSS) there was some indication that Bt BEVs improved survival, weight loss, disease activity and increased IL-10 production. Pre-treatment (conditioning) of murine bone marrow derived monocytes (BMDM) with Bt BEVs resulted in higher ratio of IL-10/TNFα production after an LPS challenge when compared to LPS pre-conditioned or non-conditioned BMDM. Using the THP-1 monocytic cell line the interactions between Bt BEVs and monocytes/macrophages were shown to be mediated primarily by TLR2. Histone (H3K4me1) methylation analysis showed that Bt BEVs induced epigenetic reprogramming which persisted after infectious challenge, as revealed by increased levels of H3K4me1 in Bt BEV-conditioned LPS-challenged BMDM. Collectively, our findings highlight the important role of Bt BEVs in maintaining host immune homeostasis and raise the promising possibility of considering their use in immune therapies.

## Introduction

The ecosystem of the human gastrointestinal tract (GIT) is shaped by complex interactions between resident microbes (the microbiota), the epithelium and immune cells. Host–microbe interactions have traditionally been analyzed from the perspective of pathogenic relationships, but it has become evident that commensal microbes also exert important beneficial effects on the host. The ability of immune cells to discriminate between pathogens and commensal bacteria is therefore essential to maintain immune homeostasis and preserve host health, by simultaneously providing protection against pathogens and tolerance toward symbiotic microbiota (Bron et al., 2011).

Innate immunity plays an important role in intestinal protection and is the first line of host defense against infection comprising physical, chemical, and cellular barriers. Various stimuli and conserved microbe-associated molecular pattern (MAMPs) molecules can activate and modulate innate immunity and inflammatory responses with enhanced or decreased production of pro-inflammatory mediators and cytokines depending on the type and dose of the ligand recognized by individual pattern recognition receptors (PPRs; Ifrim et al., 2014). Bacterial lipopolysaccharides (LPS), one of the major triggers of inflammatory response *via* interactions with toll-like receptor 4 (TLR4), can induce a state of tolerance in macrophages and monocytes after repeated or prolonged exposure, resulting in reduced pro-inflammatory cytokine production (Novakovic et al., 2016; Seeley and Ghosh, 2017). The innate immune system can be also activated by sterile endogenous dietary substances from Western-type diets that can contribute to various chronic inflammatory diseases (Bekkering et al., 2014; Christ and Latz, 2019). Innate immune activation leads to modifications in the chromatin state of the innate immune cells, with epigenetic changes persisting even after the cells return to homeostasis, altering their long-term responsiveness to re-infection (Netea et al., 2020). Enhanced inflammatory responses in trained innate immune cells and diminished activation in tolerized innate immune cells are based on epigenetic reprogramming events, including DNA methylation and histone modifications that up- or down-regulate the transcription of inflammatory genes (Netea et al., 2020). Identifying the receptors, signaling pathways and epigenetic modifications that induce and maintain immune tolerance is therefore important for understanding how immune tolerance contributes to a state of controlled inflammation with potential benefits for autoimmune conditions and chronic inflammation diseases without contributing to immunodeficiency.

All bacteria naturally produce and release nano-sized, non-replicative extracellular vesicles (BEVs) with roles in response to stress, quorum sensing, biofilm formation, and interspecies and interkingdom communication (Schwechheimer and Kuehn, 2015). BEVs contain various cargo including enzymes, signaling molecules, and metabolites (Bryant et al., 2017). BEVs generated by Gram-negative pathogens contain toxins and virulence factors that can breach host defenses facilitating invasion and infection by parental cells (Kaparakis-Liaskos and Ferrero, 2015). By contrast, the role of BEVs produced by commensal microbes, and in particular the vast numbers residing in the GIT, is less clear, although recent studies have identified a potential role in host-microbe communication and in maintaining immune homeostasis *via* interactions with dendritic cells (Durant et al., 2020). BEVs generated by prominent members of the microbiota such as *Bacteroides thetaiotaomicron* (Bt) can cross the epithelial barrier of the intestine and access underlying lamina propria cells and, *via* the vasculature, other organs and tissues. They mediate bacteria-host interactions which modulate the physiology of various host cells including those of the innate and adaptive immune system (Stentz et al., 2018; Jones et al., 2020; Gul et al., 2022). BEVs produced by the pathobiont *Bacteroides fragilis* have been implicated in immune homeostasis as they can mediate anti-inflammatory effects by TLR2-dependent activation of dendritic cells and the production of IL-10 by regulatory T cells (Shen et al., 2012). The inbuilt adjuvanticity and immune-potentiation properties of Bt-derived BEVs has also been exploited in drug delivery formulations and in mucosal vaccines for respiratory viruses (Carvalho et al., 2019a,b), supporting their ability to modulate host immune cell function.

Based upon the biophysical and immunological properties of BEVs generated by commensal bacteria, we have investigated the potential of Bt-derived BEVs to act *via* their interactions with innate immune cells as modulators of the immune tolerance and the inflammation response using well-established *in vivo* and *in vitro* models. Identifying the interactions between BEVs and host innate immune cells is an important step toward considering their use in immunotherapy.

## Materials and methods

### Isolation and characterization of Bt BEVs

*Bacteroides thetaiotaomicron* VPI-5482 was grown in 500 ml of Bacteroides Defined Medium (BDM4; Supplementary Table S1) at 37°C in an anaerobic cabinet. For BEVs preparations, cells were harvested after 16 h at an approximate OD_600nm_ of 2.5 corresponding to early stationary phase. BEVs were isolated following a method adapted from Stentz et al. (2022). Briefly, Bt cultures (500 ml) were centrifuged at 6000 g for 50 min at 4°C and the supernatants filtered through polyethersulfone (PES) membranes (0.22 μm pore-size; Sartorius) to remove debris and cells. Supernatants were concentrated by cross-flow ultrafiltration (100 kDa molecular weight cut-off, Vivaspin 50R, Sartorius), the retentate was rinsed once with 500 ml of PBS (pH 7.4) and concentrated to 1 ml. Further purification of BEVs was performed by fractionation of the suspension by size-exclusion chromatography using qEVoriginal 35 nm columns (Izon) according to manufacturer’s instructions. Fractions containing BEVs were combined and filter-sterilized through a 0.22 μm PES membrane (Sartorius) and the suspensions were stored at 4°C. Absence of viable microorganisms was confirmed by plate count and absence of LPS was confirmed by Limulus Amebocyte Lysate (LAL) test.

The size and concentration of the isolated Bt BEV suspension was determined using nanoparticle tracking analysis and the ZetaView PMX-220 TWIN instrument according to manufacturer’s instructions (Particle Metrix). Aliquots of BEV suspensions were diluted 1,000-to 20,000-fold in particle-free water for analysis. Size distribution video data were acquired using the following settings: temperature: 25°C; frames: 60; duration: 2 s; cycles: 2; positions: 11; camera sensitivity: 80 and shutter value: 100. The ZetaView NTA software (version 8.05.12) was used with the following post acquisition settings: minimum brightness: 20; max area: 2,000; min area: 5 and trace length: 30.

### Animal studies

Specific-pathogen-free (SPF) C57BL/6 male mice were bred and maintained in the Disease Modeling Unit at the University of East Anglia (United Kingdom). Animals were housed in individually ventilated cages and exposed to a 12 h light/dark cycle with free access to drinking water and standard laboratory chow diet. Animal experiments were conducted in full accordance with the Animal Scientific Procedures Act 1986 under UK Home Office (HMO) approval and HMO project license 70/8232.

### Acute colitis mouse model

The dextran sulfate sodium (DSS) induced mouse model of acute colitis was used to investigate the therapeutic potential of BEVs on intestinal inflammation. SPF C57BL/6 mice, 8 weeks old, were divided into two groups and administered with either PBS (*n* = 8) or BEVs (*n* = 6). Experimental colitis was induced by administration of 2.25% w/v DSS (36,000–50,000 Da, MP Biomedicals) in drinking water *ad libitum* for 5 days. From day 5 until the end of the experiment, DSS was replaced by fresh water. PBS and BEVs were administered by oral gavage (100 μl at 10^11^ BEVs/ml) on days 5, 7, and 9, and on day 11 mice were euthanized by cervical dislocation after exposure to rising concentrations of CO_2_. Distal colon tissue samples (1 cm) and spleens were collected for cytokine production analysis. The extent of colitis was evaluated using survival rate and a disease activity index (Supplementary Table S2) comprising daily body weights, stool consistency, tissue and content appearance, and bleeding assessments.

Each distal colon tissue sample was cut open longitudinally and incubated in flat-bottomed 24-well plates with 0.5 ml per well of complete RPMI medium (RPMI-1640 (Sigma-Aldrich) supplemented with 10% heat-inactivated fetal bovine serum (FBS; Biosera) and 1% Pen/Strep (Sigma-Aldrich)) for 24 h in a CO_2_ incubator. Spleens were macerated in a cell strainer at 70 μm, washed with complete RPMI medium and incubated with Ammonium-Chloride-Potassium (ACK) lysis buffer (Gibco) for 10 min to lyse red blood cells. Splenocyte count was adjusted to 5×10^6^ cells/ml and incubated in flat-bottomed 96-well plates with 0.2 ml per well of complete RPMI medium, with or without restimulation with 10^9^ BEVs/ml, for 72 h at 37°C and 5% CO_2_ in a humidified incubator. Supernatants from colon and splenocyte cultures were then centrifuged and store at −80°C prior to cytokine analysis.

### Murine bone marrow-derived monocyte cultures

SPF C57BL/6 mice, 13–16 weeks old, were euthanized by cervical dislocation after exposure to rising concentrations of CO_2_. Femurs were immediately removed and placed into cold sterile PBS. Bone marrow cell suspensions were isolated by flushing the femurs and tibias with RPMI-1640 supplemented with 10% heat-inactivated FBS and 1% Pen/Strep under sterile conditions. Debris was removed by passing the suspension through a 70 μm cell strainer. Cells were washed with complete RPMI media and concentration was adjusted to 6 × 10^6^ cells/ml. Cells were seeded on flat-bottomed 12-well plates (1.2 ml/well) and incubated at 37°C and 5% CO_2_ in a humidified incubator.

### BMDM—Bt BEVs co-culture

Bone marrow-derived monocyte (BMDM) cells (1.2 ml at 6 × 10^6^ cells/ml) were incubated in flat-bottomed 12-well plates in complete RPMI medium for 24 h at 37°C and 5% CO_2_ in a humidified incubator, in the presence of either different concentrations of Bt BEVs (3 × 10^9^, 3 × 10^7^, and 3 × 10^5^ BEVs/ml), LPS from *E. coli* (10 ng/ml; Sigma-Aldrich) as a positive control or PBS as negative control. After 24 h, supernatants were collected and stored at −20°C prior to cytokine measurements. Cells were washed with warm PBS and maintained in complete RPMI medium for 5 days at 37°C and 5% CO_2,_ with fresh media added on day 3. Cells were then incubated with LPS (10 ng/ml) to mimic an infectious challenge, or PBS as negative control. After 24 h, supernatants were collected and stored at −20°C for subsequent cytokine measurement. Cells were washed with warm PBS and detached from the wells by gently scraping after 1 h incubation with ice-cold Macrophage Detachment Solution (PromoCell) at 4°C. Cells were then washed with PBS containing 0.5 mM EDTA and stored at −80°C in FBS with 10% DMSO (Sigma-Aldrich) for histone methylation analysis. All incubations were performed in triplicate in two independent experiments.

### Cytokine measurements

The production of IL-10 by colon tissue and splenocytes, and TNFα, IL-6, and IL-10 produced by BMDM after 24 h of conditioning and after 24 h of challenge was measured by ELISA (Invitrogen) according to the manufacturer’s instructions. The results were recorded as pg of each cytokine per mL of supernatant.

### THP1-Blue cell assay

THP1-Blue NF-κB cells (Invivogen) were derived from the human THP-1 monocyte cell line by stable integration of an NF-κB-inducible secreted alkaline phosphatase (SEAP) reporter construct. THP1-Blue cells were cultivated in RPMI-1640 supplemented with 10% heat-inactivated FBS, 1% Pen/Strep and 100 μg/ml Normocin (Invivogen) at 37°C and 5% CO_2_ in a humidified incubator. To maintain selection pressure during cell subculturing, 10 μg/ml blasticidin was added to the growth medium every other passage.

To establish the threshold dose of BEVs that allows for the quantification of THP-1 cells activation, cells were seeded in flat-bottomed 96-well plates at a density of 5 × 10^5^ cells/ml and incubated for 24 h at 37°C and 5% CO_2_ in a humidified incubator in the presence of different concentrations of Bt BEVs (from 3 × 10^9^ to 3 × 10^6^ BEVs/ml) using LPS (10 ng/ml) as a positive control and BDM4 and PBS as negative controls.

To identify pattern recognition receptors (PRRs) involved in BEV-mediated THP1-Blue cell activation, cultures were incubated with different inhibitors of TLR2 (PAb-hTLR2 (5 μg/ml), Invivogen), TLR4 (PAb-hTLR4 (5 μg/ml), Invivogen), NOD1 (ML130 (5 μg/ml), Abcam), and NOD2 (GSK717 (5 μg/ml), Merck) prior to the addition of BEVs (3×10^8^/ml). Heat-killed *Listeria monocytogenes* (HKLM, 10^7^ cells/ml, Invivogen), LPS (10 ng/ml, Sigma-Aldrich), lauroyl-g-D-glutamyl-meso-diaminopimelic acid (DAP, 1 μg/ml, Invivogen) and N-acetylmuramyl-L-alanyl-D-isoglutamine (MDP, 10 μg/ml, Invivogen) were used as specific ligands for each inhibitor, respectively. Subsequently, 20 μl of the cell suspension was added to wells of 96-well plate, mixed with 180 μl of Quanti-Blue (Invivogen) colorimetric assay reagent and incubated for 1 h at 37°C to allow color development. NF-κB-inducible SEAP levels were quantified by absorbance reading at 620 nm. All incubations were performed in triplicate in three independent experiments.

### THP1-Blue cell—Bt BEVs co-culture

To investigate phenotypic changes in THP1-Blue cells after exposure to with Bt BEVs, the cells (1.2 ml at 10^6^ cells/ml) were incubated on flat-bottomed 12-well plates in complete RPMI medium for 24 h at 37°C and 5% CO_2_ in a humidified incubator, in the presence of different concentrations of Bt BEVs (5 × 10^8^ BEVs/ml, 5 × 10^6^ BEVs/ml, and 5 × 10^4^ BEVs/ml), using LPS from *E. coli* (10 ng/ml; Sigma-Aldrich) as a reference and positive control and PBS as a negative control. Cells were washed with warm PBS and detached from the wells by gently scraping after 1 h incubation with ice-cold Macrophage Detachment Solution at 4°C. Cells were then washed with PBS containing 0.5 mM EDTA and collected for flow cytometry analysis.

### Flow cytometry

THP1-Blue cells were stained with Zombie Aqua Fixable Viability kit (BioLegend) following the manufacturer’s protocol. Cells were washed with Cell Staining Buffer (BioLegend) before blocking Fc receptors by incubation with Human TruStain FcX (BioLegend) for 10 min at 21°C. Cells were then surface stained for 20 min at 4°C in the dark using APC/Fire 750 anti-human CD14 Clone 63D3 (BioLegend) and flow cytometry performed on BD LSRFortessa Cell Analyzer (BD Biosciences) with the data analyzed using FlowJo software 10.8.1 (BD Biosciences).

### Histone methylation

Histone proteins from bone marrow-derived cells were extracted using the EpiQuik Total Histone Extraction Kit (Epigentek) according to the manufacturer’s instructions and quantified by measuring absorbance at 280 nm. The level of histone 3 lysine 4 mono-methylation (H3K4me1) was quantified using the ELISA-based colorimetric kit EpiQuik Global Mono Methyl Histone H3 K4 Quantification (Epigentek) and results were expressed as ng of H3K4me1 per μg of total protein.

### Statistical analysis

*In vitro* data were subjected to One-way ANOVA or Two-way ANOVA followed by Tukey’s multiple comparison *post-hoc* test or Dunnett’s multiple comparison *post-hoc* test using GraphPad Prism 5 software. Data are presented as the mean ± standard deviation. *In vivo* results were compared using Mann-Whitney *U*-tests (for independent groups) or log-rank tests for survival data. These were conducted using R version 4.2.0. Statistically significant differences between two mean values were established by a *p* < 0.05.

## Results

### Anti-inflammatory effect of Bt BEVs *in vivo*

**Figure 1:**
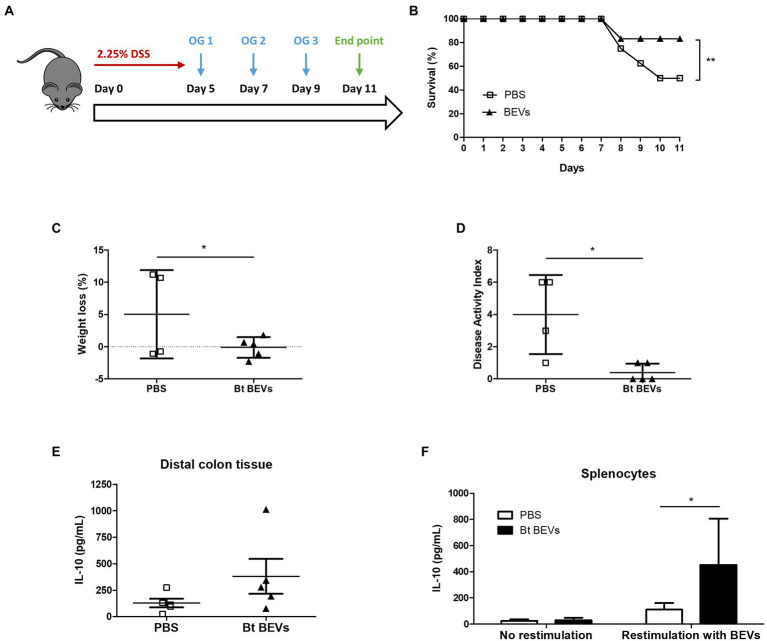
Bt BEVs ameliorate DSS-induced colitis in mice. **(A)** Mice were provided with drinking water containing 2.25% DSS (w/v) for 5 days. On days 5, 7, and 9 mice were orally administered with either PBS or Bt BEVs (100 μl at 10^11^ BEV/ml). **(B)** Survival rates. **(C)** Percent weight loss at day 11. **(D)** Disease Activity Index (DAI) at day 11. **(E)** IL-10 production in distal colon tissue. **(F)** IL-10 production by splenocytes cultured in complete media alone or in media containing Bt BEVs. Graphs depict mean ± SD values. ^*^p < 0.05, ^**^p < 0.01.

To investigate the ability of Bt BEVs to influence inflammatory responses *in vivo* we used DSS-induced murine colitis as a model of acute inflammation (Figure 1A). This is a well-established lymphocyte-independent model of intestinal inflammation in which the clinical severity can be quantified, providing a reliable method to study the contribution of the innate immune system to inflammatory responses in the host. Mice orally administered with Bt BEVs exhibited a significantly higher (*p* < 0.01) survival rate throughout the experiment compared to mice that received vehicle (PBS) only (Figure 1B). Oral administration of Bt BEVs also contributed to a significant decrease (*p* < 0.05) in weight loss and disease activity index scores (Figures 1C,D). Analysis of cytokine production by freshly excised and cultured colonic tissue showed that production of the anti-inflammatory cytokine IL-10 was higher in distal colon tissue from mice previously administered with Bt BEVs (Figure 1E). When splenocytes from mice orally gavaged with Bt BEVs were restimulated *ex vivo* with Bt BEVs, IL-10 production was also significantly increased (*p* < 0.05). By comparison, there was no difference in IL-10 production from non-stimulated splenocytes across all experimental groups (Figure 1F).

### Bt BEVs modulation of cytokine production in BMDM

**Figure 2:**
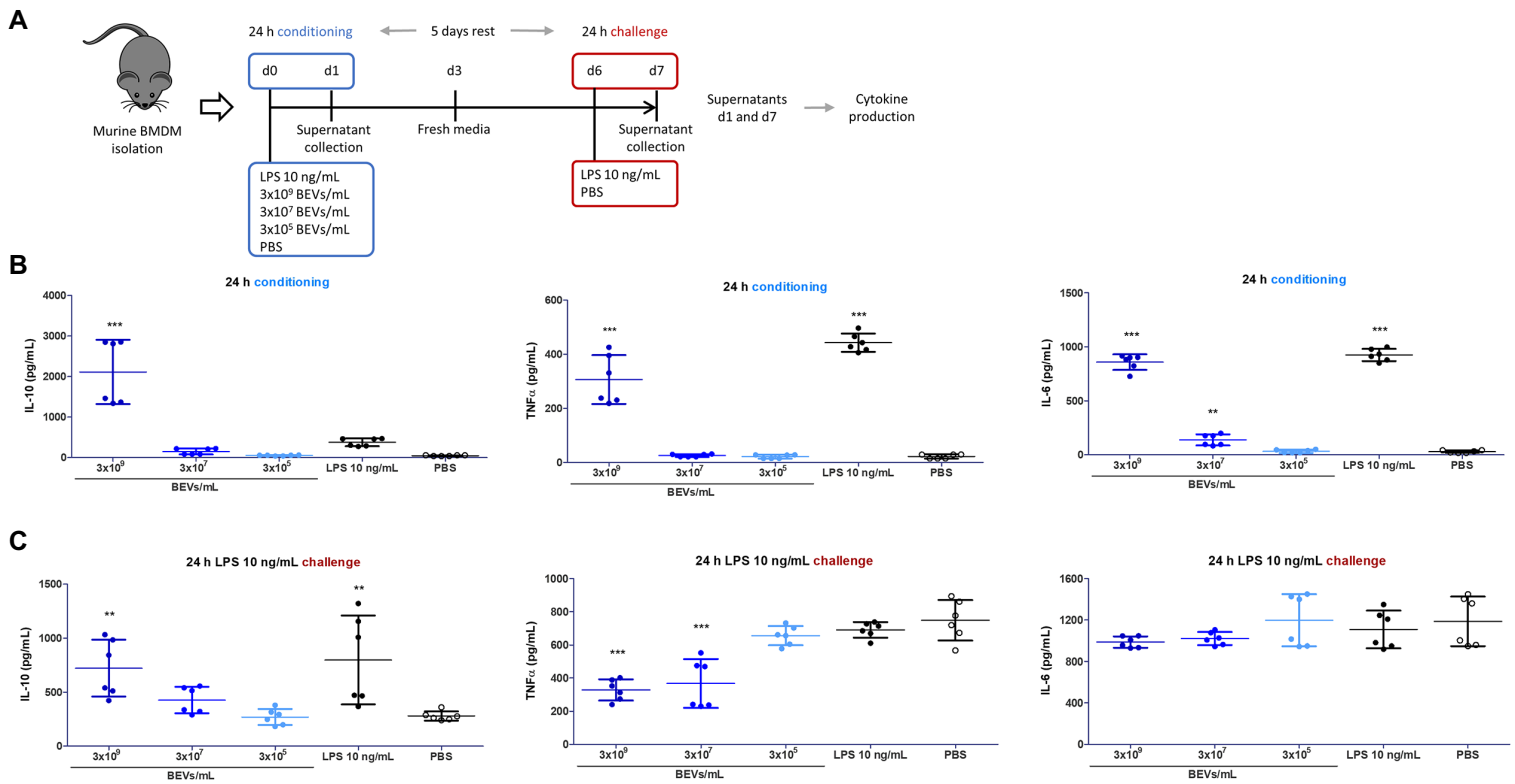
Bt BEVs modulate the production of anti- and pro-inflammatory cytokines by murine bone marrow derived macrophages (BMDM). **(A)** Experimental plan. **(B)** Cytokine production by BMDM 24 h after conditioning with Bt BEVs and LPS was assessed by ELISA. **(C)** Cytokine production by Bt BEV- and LPS-conditioned BMDM 24 h after exposure to LPS (10 ng/ml) determined by ELISA. Non-conditioned BMDM (PBS) were used as the reference group for statistical analysis. Graphs depict mean ± SD values. ^**^p < 0.01; ^***^p < 0.001.

Bone marrow mononuclear cells cultured under conditions that favor the growth of monocytes and macrophages were used to examine further the potential interaction of Bt BEVs with innate immune cells. Specifically, we investigated how Bt BEVs influence murine bone marrow-derived monocytes (BMDM) in response to an inflammatory stimulus. Cytokine production in BEV-conditioned BMDM was measured before and after an infection-like challenge with LPS (Figure 2A). We observed significantly increased amounts (*p* < 0.001) of IL-10 in BMDM cultures after incubation for 24 h with 3 × 10^9^ BEVs/ml. No significant changes in IL-10 production were detected in BMDM containing lower concentrations of Bt BEVs or with LPS and PBS. The concentrations of the pro-inflammatory cytokines TNFα and IL-6 were significantly higher (*p* < 0.001) in BMDM incubated with 3 × 10^9^ BEVs/ml when compared to PBS alone. However, these levels were lower than in LPS-conditioned BMDM cultures (Figure 2B). Analysis of cytokine production in BEV-conditioned BMDM cultures after an LPS challenge revealed opposite trends when comparing anti- and pro-inflammatory activity. While IL-10 production was directly associated with the Bt BEVs concentration used to condition BMDM, the levels of pro-inflammatory cytokines showed an inverse relationship with the Bt BEV conditioning dose, with TNFα production significantly decreased (*p* < 0.001) when BMDM were incubated with BEVs at 3 × 10^7^ BEVs/ml or higher concentrations (Figure 2C). Collectively, these data show that Bt BEVs exert in a dose-dependent manner anti-inflammatory responses from monocytes/macrophages, which was particularly noticeable in a re-infection (LPS challenge) scenario.

### Molecular basis of BEV-monocyte interactions and the involvement of specific pattern recognition receptors

**Figure 3:**
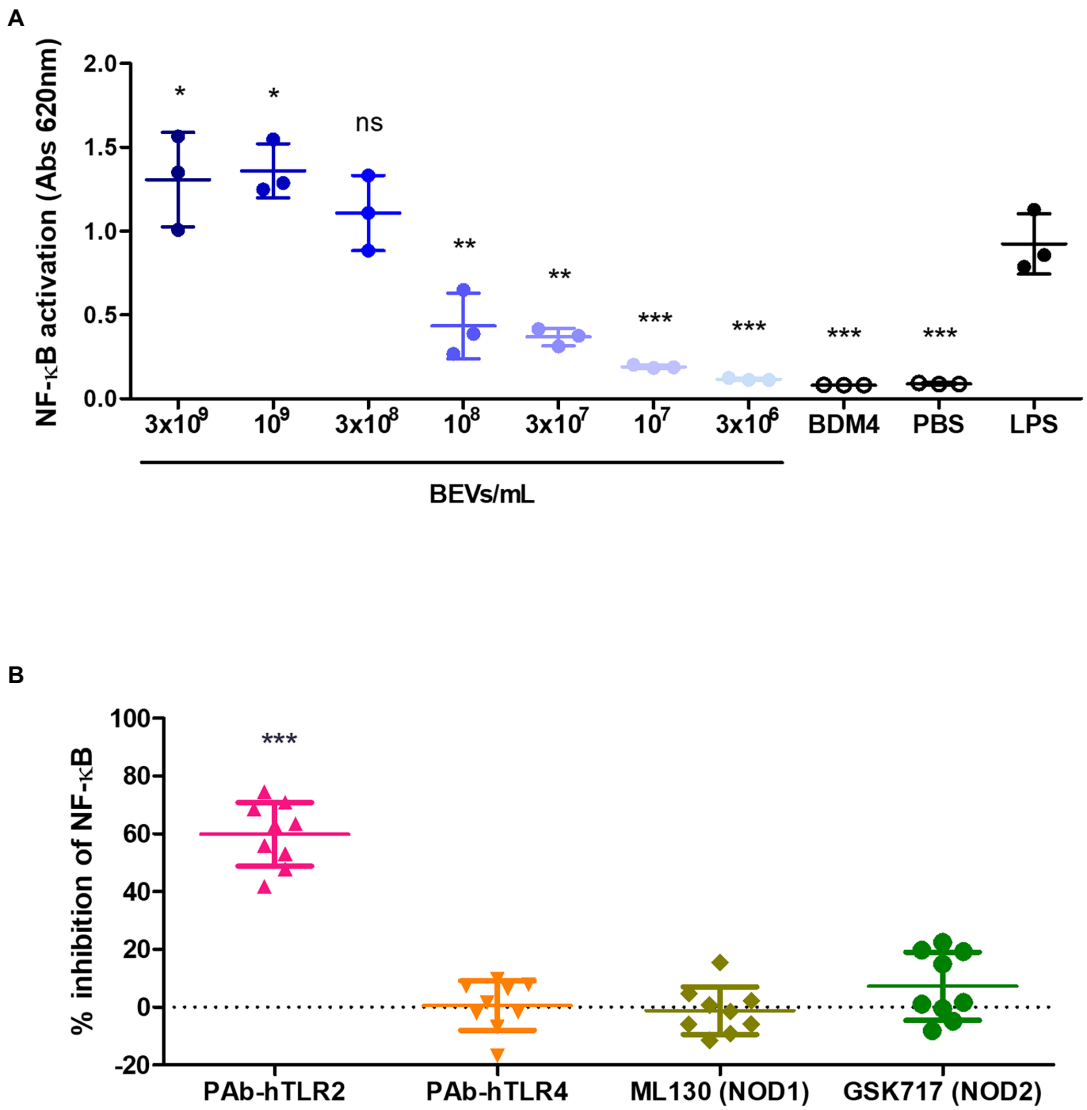
Mediators of Bt BEV-monocyte/macrophage interaction identified using THP1-Blue monocytes. **(A)** THP-1 cells were incubated with a range of Bt BEV concentrations or with Bt growth media (BDM4), PBS or LPS for 24 h prior to assessing level of NF-kB activation. Statistical analysis was performed using LPS as the reference group. **(B)** THP-1 cells were pre-incubated with optimal concentrations of TLR2, TLR4, NOD1 or NOD2 inhibitors prior to the addition of Bt BEVs and subsequent assessment of NF-kB activation. Graphs depict mean ± SD values. ns: *p* > 0.05; ^*^p < 0.05; ^**^p < 0.01; ^***^p < 0.001.

The human monocytic cell line THP-1 that expresses an NF-κB inducible secreted alkaline phosphatase (SEAP) reporter construct (THP1-Blue) was used to identify the receptors and pathways in monocytes involved in the anti-inflammatory effect of Bt BEVs observed in both the *in vivo* and *in vitro* model systems. To investigate if Bt BEVs can activate THP-1 cells and to establish the activation threshold, cells (5 × 10^5^ cells/ml) were incubated with different concentrations of Bt BEVs (3 × 10^6^ BEVs/ml to 3 × 10^9^ BEVs/ml) using a chemically defined Bt media (BDM4) and PBS or LPS (10 ng/ml) as negative and positive controls, respectively. NF-κB activation in THP-1 cells in response to Bt BEVs was dose-dependent (Figure 3A) with no detectable activation seen in cultures containing BDM4 or PBS alone. Based upon the level of activation induced by 3 × 10^8^ BEVs/ml being equivalent (*p* > 0.05) to that of LPS (10 ng/ml), we established this as an optimal Bt BEVs concentration for subsequent inhibition assays. Specific inhibitors of key extracellular and intracellular PRRs were used in THP1-BEV co-cultures to identify those contributing to NF-κB activation in THP-1 cells. In a series of optimization experiments, the optimal concentration of each inhibitor was established by titration and their specificity at that concentration was confirmed with individual PRR-specific ligands (Supplementary Figure S1). The most potent inhibition of NF-κB activation (~60%) was seen in cultures containing PAb-hTLR2 (5 μg/ml), an antibody that specifically inhibits TLR2 activation (Figure 3B; Supplementary Figure S1), consistent with TLR2 activation mediating interactions between Bt BEVs and monocyte/macrophages (Figure 3B). By contrast, no significant inhibition of NF-κB activation was seen using inhibitors of TLR4, NOD1, or NOD2 (Figure 3B).

### Phenotypic changes in monocytes following stimulation with Bt BEVs

**Figure 4:**
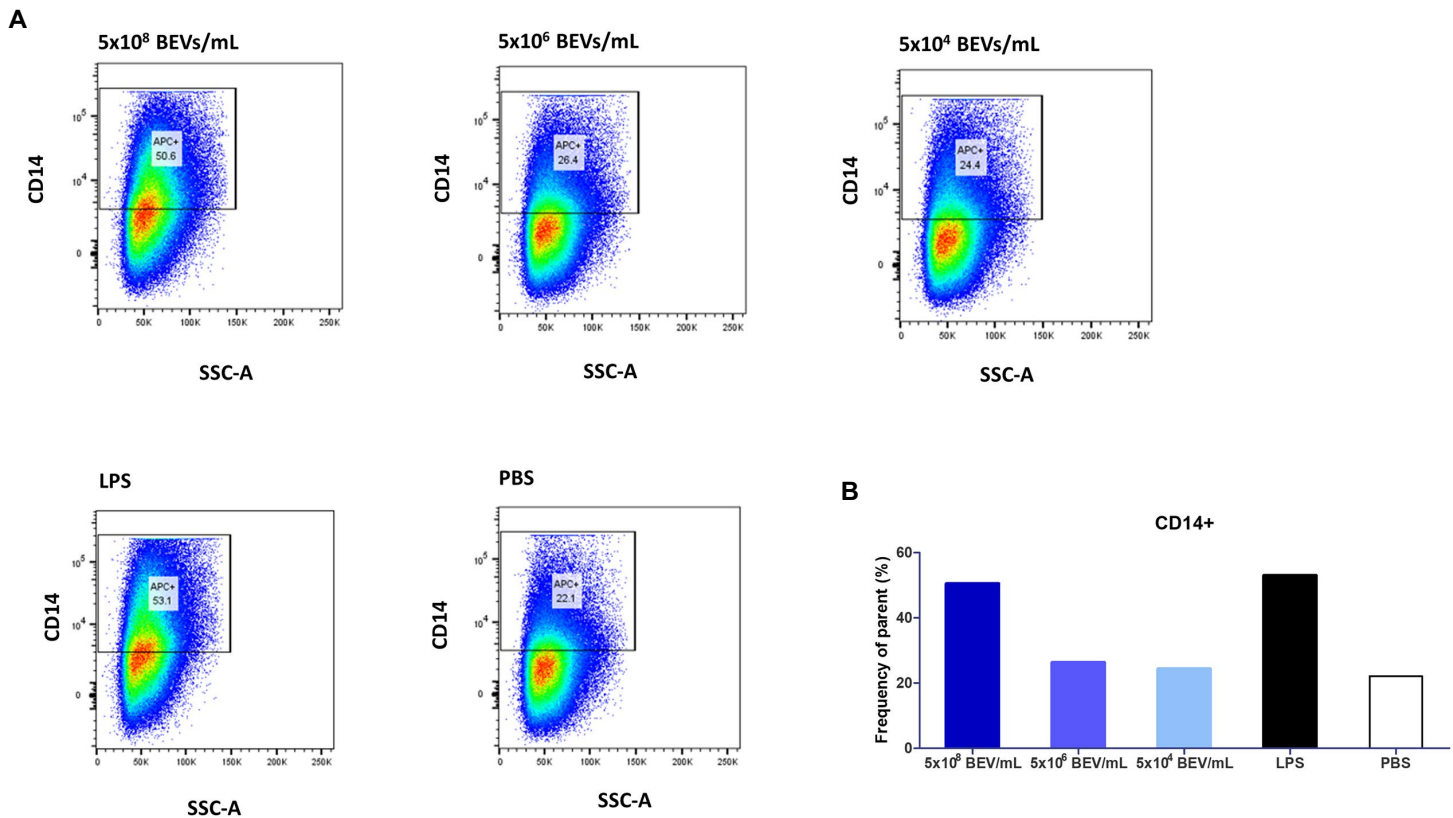
Bt BEVs elicit phenotypic changes in THP1-Blue NF-κB cells (10^6^ cells/ml). **(A)** THP-1 cells were incubated with different concentrations of Bt BEVs for 24 h and analyzed for CD14 expression by flow cytometry. Gating strategy was based on scatterplots of CD14 vs. side scatter signal. **(B)** The bar graph represents THP-1 cells with high expression of CD14 as percentage frequency of parent.

We investigated the phenotypic changes in THP-1 cells after exposure to Bt BEVs by measuring levels of CD14 expression using flow cytometry. CD14^+^ cells were gated based on a fluorescence minus one (FMO) strategy. CD14 expression is a marker of activation related to pro-inflammatory monocytes, is a co-receptor for TLR4 responsivity to LPS, and contributes to the activation of other PRRs including TLR2. Among THP-1 cells cultured in media alone approximately 22% were CD14^+^ (Figure 4). In cultures containing the highest concentration of Bt BEVs (5×10^8^ BEV/mL equivalent to a ratio of 500 BEVs,THP-1) the proportion of CD14^+^ cells more than doubled to approximately 51% and was equivalent to the levels seen in cultures containing LPS (~53%; Figure 4). By contrast, THP-1 cells stimulated with lower concentrations of Bt BEVs showed no significant changes in the proportion of CD14^+^ monocytes when compared to cells incubated with PBS (Figure 4).

### Effect of Bt BEVs on histone modifications in BMDM

**Figure 5:**
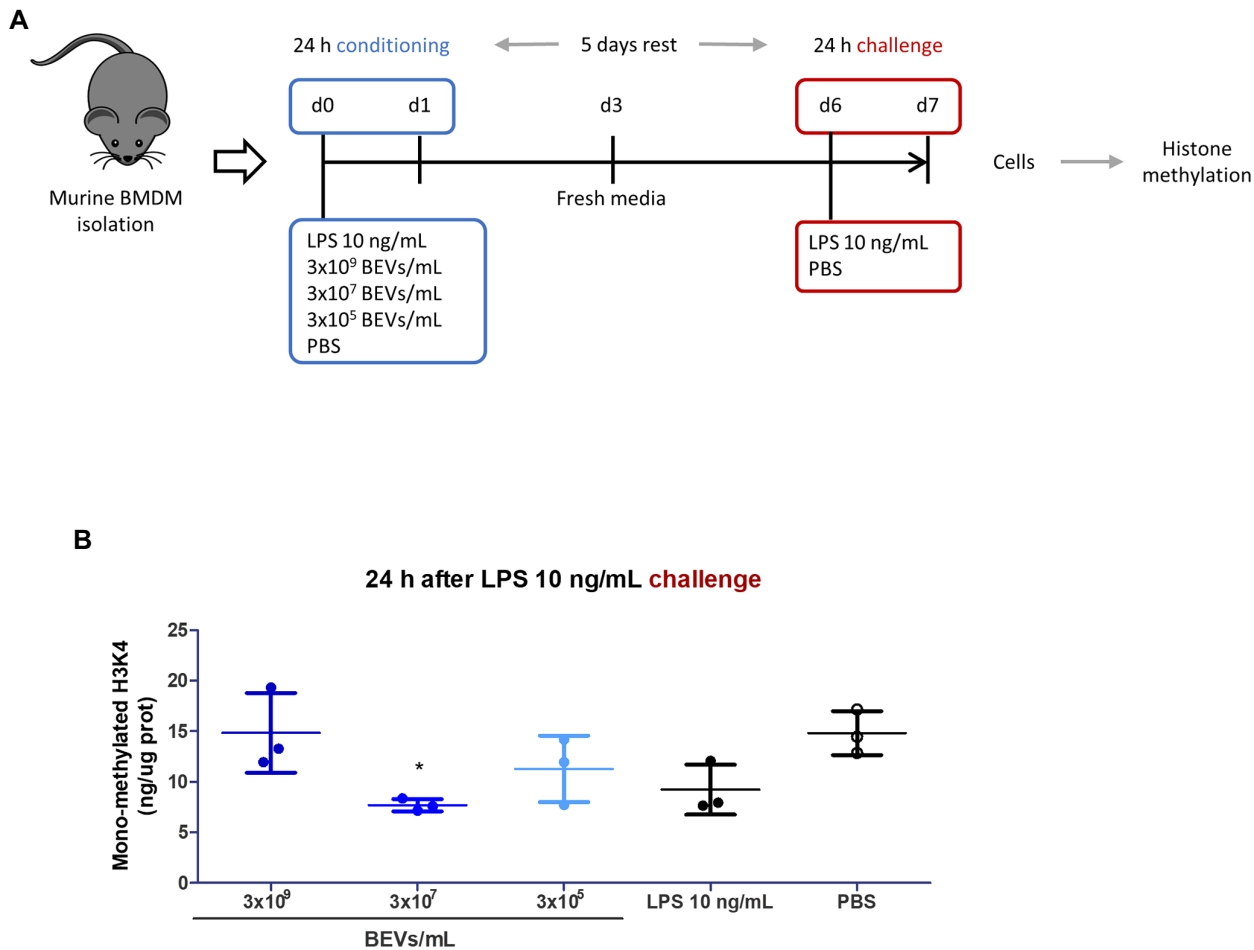
Histone mono-methylation (H3K4me1) as an epigenetic signature of Bt BEV modulation of innate immunity. **(A)** Experimental plan. **(B)** Methylation of histone proteins from Bt BEV- or LPS-conditioned BMDM 24 h after exposure to LPS (10 ng/ml) was quantified using an ELISA-based colorimetric kit and results were expressed as ng of H3K4me1 per μg of total protein. Non-conditioned BMDM (PBS) were used as the reference group for statistical analysis. Graph depicts mean ± SD values. ^*^p < 0.05.

Inflammatory processes and innate immunity are tightly regulated by epigenetic mechanisms with genomic DNA methylation and modification of histones influencing the function of innate immune cells (Saeed et al., 2014; Fraschilla et al., 2022). To determine if Bt BEVs can induce epigenetic changes, and in particular histone methylation, we measured levels of H3K4me1 in BMDM, which indicates the mono-methylation at the 4th lysine residue of histone H3 protein and is an enhancer signature (Figure 5A). Varying levels of H3K4me1 were detected in BMDM after culture with Bt BEVs, with the highest levels seen in cultures containing 3 × 10^9^ BEV/ml, comparable to those in BMDM cultured in media alone (PBS) and higher than that in cultures containing LPS (Figure 5B). By comparison, lower levels of H3K4me1 were seen in cultures containing fewer Bt BEVs (3 × 10^5^ and 3 × 10^7^ BEV/ml) with the differences being significant (*p* < 0.05) for BMDM cultured with 3 × 10^7^ BEV/ml. These data demonstrate that Bt BEVs altered the histone methylation status in innate immune cells.

## Discussion

BEVs produced by gut bacteria can cross the intestinal epithelium, gaining access to host cells (Stentz et al., 2018; Jones et al., 2020) and contribute to immune homeostasis *via* interactions with innate immune cells (Maerz et al., 2018; Diaz-Garrido et al., 2019; Durant et al., 2020). However, the details and nature of this BEV-immune cell crosstalk is incomplete. In this study, we have provided *in vitro* and *in vivo* evidence for the anti-inflammatory and immunomodulatory properties of BEVs produced by the major human gut commensal bacterium Bt and identified the molecular basis of their interaction with monocytes and macrophages. We recognize that different types of particles could be copurified with BEVs due to technical limitations but their effect is taken into consideration and appropriate controls were utilized wherever possible to aid data interpretation (Juodeikis and Carding, 2022).

Oral administration of Bt BEVs ameliorates DSS-induced colitis in mice, underlining their potential as a treatment for non-infectious autoimmune pathologies. Similar protective effects have been reported in DSS models after treatment with fresh and lyophilized cultures of Bt (Delday et al., 2019) and other *Bacteroides* species (Hudcovic et al., 2009; Chiu et al., 2014; Chang et al., 2017), although the mediators of these effects and the possibility that it includes BEVs were not investigated. The size, stability, and non-replicative status of Bt BEVs makes them good candidates for therapeutic interventions compared to whole bacteria. Conditions involving chronic inflammation of the gut are associated with dysregulation of mucosal innate immune response (Xu et al., 2014), increased NF-κB activation (Schreiber et al., 1998) and increased levels of pro-inflammatory cytokines such as TNF-α and IL-6 (Atreya et al., 2000; Komatsu et al., 2001). In this context, the significance of BEV-elicited IL-10 from BMDM is implied from its role in the prevention of inflammatory bowel disease (Mazmanian et al., 2008; Hansen et al., 2009). Mice lacking IL-10 or IL-10 receptor genes spontaneously develop intestinal inflammation (Kuhn et al., 1993) with IL-10 acting by suppressing antigen presentation by downregulating MHC class II expression (Koppelman et al., 1997) and inhibiting pro-inflammatory cytokine synthesis by blocking the activation of the inhibitor of NF-κB kinase (IKK) and dysregulating NF-κB (Schottelius et al., 1999). The beneficial effects of BEVs or Bt (Li et al., 2021) in DSS-colitis are associated with increased production of IL-10 in serum, colonic tissue and by peripheral splenocytes which can promote a non-inflammatory status by counteracting pro-inflammatory responses. The contribution of Bt BEVs to the maintenance of immune homeostasis by promoting IL-10 production by innate immune cells is also implied by our previous study in which we reported the absence of Bt BEV-elicited IL-10 production by innate immune cells isolated from patients with inflammatory bowel disease (Durant et al., 2020). It is interesting to note that lower levels of *Bacteroides* spp. are present in the gut microbiota of inflammatory bowel disease patients (Zhou and Zhi, 2016). While the present study focused on BEVs, we cannot exclude the possibility for other cell-associated or secreted constituents of Bt to contribute to immunoregulatory responses *in vivo*. Indeed, administration of live or dead (freeze dried) Bt to IL-10r-deficient mice protects the animals from developing colitis (Delday et al., 2019) which may involve different bacterial mediators and multiple interactions with host cells. Nevertheless, Bt and BEV-elicited IL-10 production by host immune cells appears to be central to their protective effects.

In BMDM cultures pre-conditioned with BEVs prior to an infection-like challenge with LPS, high doses of Bt BEVs significantly upregulated the production of IL-10. Although Bt BEVs also increased the production of pro-inflammatory cytokine TNFα in BMDM, the levels were significantly lower than those achieved with LPS. This could be explained by the inhibitory effect of IL-10 on pro-inflammatory cytokine synthesis. This anti-inflammatory effect was more evident in a subsequent LPS-challenge. Pre-conditioning by Bt BEVs altered the cytokine profile of BMDM in a dose-dependent manner, especially in the case of the IL-10/TNFα ratio. High doses of Bt BEV pre-conditioning produced a high IL-10/TNFα ratio, indicatory of a homeostatic or tolerance like status and an attenuated inflammatory response to LPS stimulation. This phenomenon resembles that of endotoxin tolerance which is characterized by upregulated IL-10 and downregulated TNFα production leading to an immune hyporesponsiveness (Biswas and Lopez-Collazo, 2009; Gu et al., 2022). Interestingly, IL-6 levels were not affected by BEV conditioning which was also noted in a related study investigating the immunomodulatory effect of different *Bacteroides* species on murine bone marrow-derived dendritic cells (BMDC; Steimle et al., 2019). In this study, IL-6 secretion was also not reduced in *Bacteroides*-primed and *E. coli*-challenged BMDC, and priming of BMDC with *Bacteroides* resulted in decreased TNFα expression after *E. coli* challenge in contrast to non-primed BMDCs. Since IL-6 is a pleiotropic cytokine capable of acting as a defense mechanism in acute inflammation, and conversely exhibits a pro-inflammatory profile in chronic inflammation (Scheller et al., 2011), further studies are required to determine the significance of IL-6 production in BMD-innate immune cells conditioned with BEVs. It is also interesting to note the contrasting impacts of intact *Bacteroides* cells versus their BEVs on cytokine production. Whereas we have identified and confirmed IL-10 production as a signature of BEV interaction with human (Durant et al., 2020) and murine innate immune cells, this signature is not as evident using intact *Bacteroides* cells as a stimulus, suggesting that commensal gut bacteria can utilize different means including both cell-associated and secreted mediators to communicate with and influence host immune cells.

The molecular basis of Bt BEV-monocyte interactions was established using the human monocytic reporter cell line THP1-Blue NF-κB. TLR2 activation was shown to mediate Bt BEV-elicited NF-κB activation, whereas TLR4, NOD1, and NOD2 made no significant contribution. In a previous study (Gul et al., 2022), we reported the influence of TLR2 in Bt BEV-host communication *via* the TLR2/TLR4 adaptor protein TIRAP (toll-interleukin-1 receptor domain-containing adaptor protein), although TLR4 alone also showed some involvement. This apparent discrepancy is most likely explained by our prior use of a complex bacteria growth media (Brain-Heart Infusion, BHI) containing animal tissue and cellular lipids which may function as TLR4 ligands. This factor was excluded in this study by the use of a chemically defined media (BDM4). TLR2 recognizes several microbial products from both Gram-positive and Gram-negative bacteria, including lipoproteins and peptidoglycans, and forms heterodimers with either TLR1 or TLR6 for downstream signaling *via* NF-κB pathway (Akira and Takeda, 2004). The polysaccharide A (PSA) antigen expressed in BEVs from the closely related commensal *Bacteroides fragilis* also interact with dendritic cells in a TLR2-dependent manner (Round et al., 2011; Shen et al., 2012). It has been recently reported the presence of serine-dipeptide lipids in Bt BEVs (Sartorio et al., 2022), which can also act as TLR2 ligands (Clark et al., 2013; Nemati et al., 2017). Further detailed biochemical characterization of BEV-associated lipoproteins is required to identify the ligands triggering TLR2 signaling pathways in innate immune cells. TLR2 signaling has been reported to have a protective role in inflammatory conditions (Lowe et al., 2010; Brun et al., 2013) and colorectal cancer (Sittipo et al., 2018), and to promote immune homeostasis by inhibiting the expression of pro-inflammatory cytokines and enhancing IL-10 production (Chang et al., 2017). These findings align with our proposal that the anti-inflammatory effect elicited by Bt BEVs is associated with modulations in the host innate immune system through the IL-10 signaling pathway, triggered by BEV-TLR2 interactions.

TLR activation depends on different co-receptors such as CD14, which is widely used as a marker of activation related to pro-inflammatory and classical monocytes (Lotz et al., 2004). Although generally characterized as a co-receptor for the TLR4 responsivity to LPS, CD14 also contributes to the activation of other PRRs including TLR2 (van Bergenhenegouwen et al., 2013). CD14 binds to triacylated lipopeptides, typically present in Gram-negative bacteria including *Bacteroides*, to enhance their recognition by the TLR2/TLR1 heterodimer (Jin et al., 2007). The highest proportion of CD14^+^ THP-1 cells were seen after stimulation with high concentrations of Bt BEVs, with levels equivalent to those stimulated with LPS, which suggests the functional involvement of CD14 in TLR2-mediated innate immune response induced by Bt BEVs.

The functional phenotype of immune cells is highly dependent on the establishment of unique epigenetic profiles that integrate microenvironmental cues into the genome to establish specific transcriptional programs (Calle-Fabregat et al., 2020). Among key epigenetic markers is the acquisition of histone 3 lysine 4 methylation (H3K4me1) in short lived monocytes and macrophages (van der Meer et al., 2015; Netea et al., 2016) and in long-lived myeloid bone marrow progenitors (Kaufmann et al., 2018; Mitroulis et al., 2018). The highest levels of H3K4me1 in Bt BEV-conditioned LPS-challenged BMDM, were evident in monocyte/macrophages incubated with high concentrations of Bt BEVs. Unexpectedly, these levels of H3K4me1 were higher than in LPS-conditioned BMDM and comparable to those found in non-conditioned BMDM. This seems to contradict our cytokine production results, since the repressed pro-inflammatory cytokine expression prompted by Bt BEV conditioning would be expected to correlate with close chromatin and low H3K4me1 levels. However, the IL-10 genomic locus of monocytes is poised for activation with open chromatin already at the steady state (Northrup and Zhao, 2011; Tamassia et al., 2013) and the presence of H3K4me1 is associated with IL-10 gene enhancers (Taubert, 2017), which could explain the increased levels of H3K4me1 found in BMDM conditioned with Bt BEVs. However, we cannot confirm this since our approach comprised global histone modifications. To further investigate the role of H3K4me1 and other relevant modifications involved in immune tolerance chromatin immunoprecipitation sequencing (ChIP-seq) could be used to localize these histone modifications throughout the genome.

## Conclusion

We have shown that BEVs from the major gut commensal bacterium Bt elicit anti-inflammatory and immunomodulatory properties in innate immune cells, consistent with promoting and maintaining host immune homeostasis. Bt BEVs alleviated acute intestinal inflammation in DSS-treated mice, in association with increased IL-10 production. This was confirmed *in vitro* with increased IL-10 and decreased TNFα production in BEV-conditioned and LPS-challenged BMDM cultures. BEV-mediated monocyte activation and cytokine production was mediated by TLR2 interactions and resulted in stable epigenetic changes reflected by increased levels of H3K4me1. These findings provide the rationale and basis for investigating the potential of Bt BEVs as an immune therapy.

## Data availability statement

The original contributions presented in the study are included in the article/Supplementary material, further inquiries can be directed to the corresponding author.

## Ethics statement

The animal study was reviewed and approved by Animal experiments were conducted in full accordance with the Animal Scientific Procedures Act 1986 under UK Home Office (HMO) approval and HMO project license 70/8232.

## Author contributions

SF and SC conceived and designed the experiments and wrote the manuscript. SF carried out the experimental work with contribution from AC, AM-C, EJ, RJ, and RS. All authors contributed to the article and approved the submitted version.

## Funding

This research was funded by the BBSRC Institute Strategic Programme Grant Gut Microbes and Health BB/R012490/1 and its constituent projects BBS/E/F/000PR10353 (Theme 1, Determinants of microbe-host responses in the gut across life) and BBS/E/F/000PR10355 (Theme 2, Changes in gut microbe-host interactions and their impact beyond the gut) and Quadram Institute Bioscience Proof-of-Concept Research Grant Fund project number 43134-000-A.

## Conflict of interest

The authors declare that the research was conducted in the absence of any commercial or financial relationships that could be construed as a potential conflict of interest.

## Publisher’s note

All claims expressed in this article are solely those of the authors and do not necessarily represent those of their affiliated organizations, or those of the publisher, the editors and the reviewers. Any product that may be evaluated in this article, or claim that may be made by its manufacturer, is not guaranteed or endorsed by the publisher.
