# Corrigendum: Extracellular vesicles produced by the human gut commensal bacterium *Bacteroides thetaiotaomicron* elicit anti-inflammatory responses from innate immune cells

**DOI:** 10.3389/fmicb.2023.1353539

**Published:** 2024-01-04

**Authors:** Sonia Fonseca, Ana L. Carvalho, Ariadna Miquel-Clopés, Emily J. Jones, Rokas Juodeikis, Régis Stentz, Simon R. Carding

**Affiliations:** ^1^Gut Microbes and Health, Quadram Institute Bioscience, Norwich, United Kingdom; ^2^Department of Women's and Children's Health, Institute of Life Course and Medical Sciences, University of Liverpool, Liverpool, United Kingdom; ^3^Norwich Medical School, University of East Anglia, Norwich, United Kingdom

**Keywords:** extracellular vesicles, Bacteroides, anti-inflammatory response, innate immune tolerance, BMDM, THP-1 cells, TLR2, IL-10

In the published article, there was an error in Figure 1 as published. Statistical significance values were incorrectly assigned to the figure panels. The corrected Figure 1 and its caption appear below.

**Figure 1:**
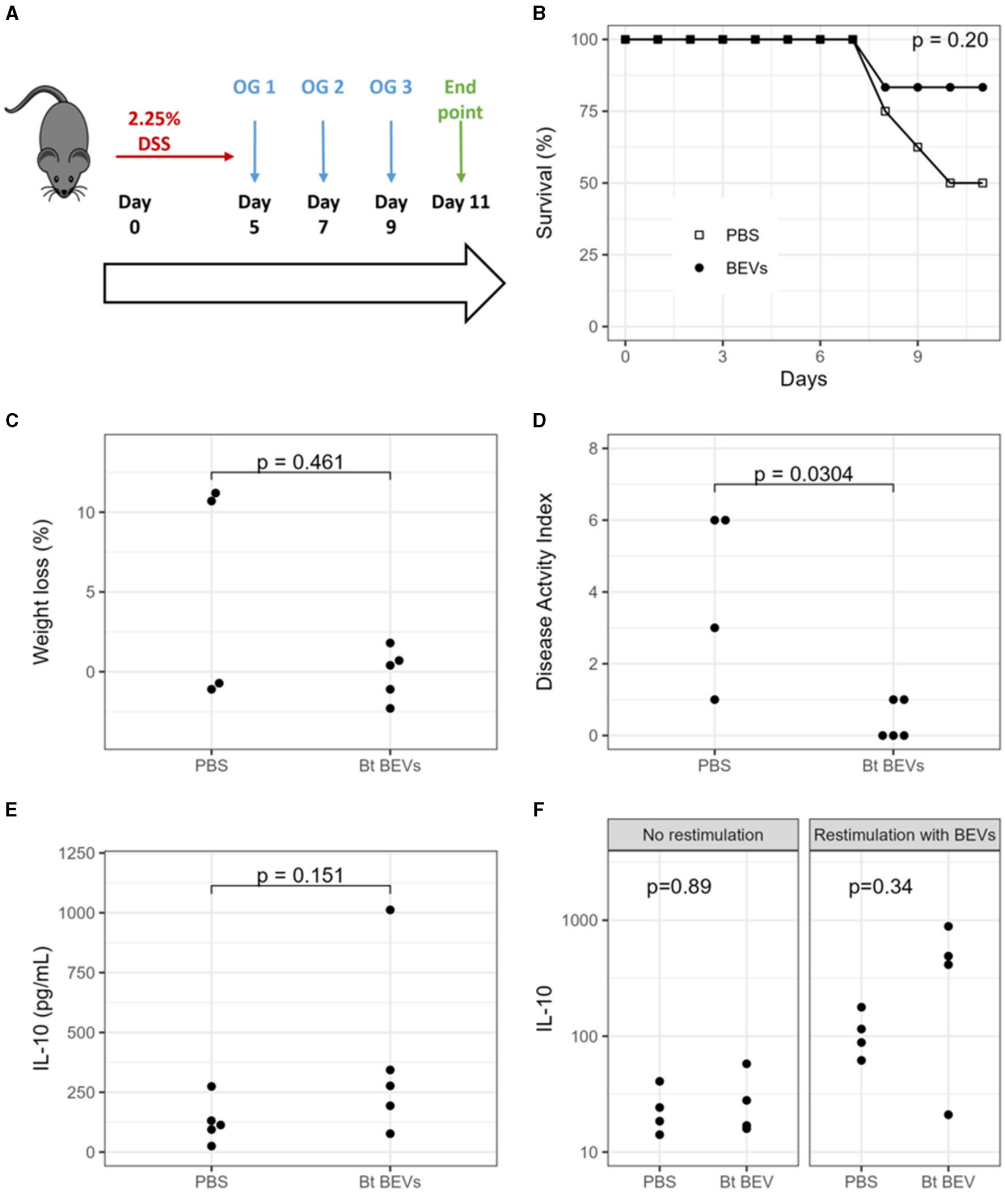
Impact of Bt BEVs on DSS-induced colitis in mice. **(A)** Mice were provided with drinking water containing 2.25% DSS (w/v) for 5 days. On days 5, 7, and 9 mice were orally administered with either PBS or Bt BEVs (100 μl at 10^11^ BEV/ml). **(B)** Survival rates. **(C)** Percent weight loss at day 11 among surviving mice. **(D)** Disease Activity Index (DAI) at day 11 among surviving mice. **(E)** IL-10 production in distal colon tissue. **(F)** IL-10 production by splenocytes cultured in complete media alone or in media containing Bt BEVs. *P*-value for panel **(A)** calculated by a log-rank test, for panels **(B–F)** calculated using Mann-Whitney *U*-tests.

In the published article, there was an error in the **Abstract**.

This previously stated:

“Administration of Bt BEVs to mice treated with colitis-inducing dextran sodium sulphate (DSS) ameliorates the symptoms of intestinal inflammation, improving survival rate and reducing weight loss and disease activity index scores, in association with upregulation of IL-10 production in colonic tissue and in splenocytes.”

The corrected sentence appears below:

“In mice treated with colitis-inducing dextran sodium sulfate (DSS) there was some indication that Bt BEVs improved survival, weight loss, disease activity and increased IL-10 production.”

In the **Materials and methods** there was an error in the section *Statistical analyses*. This previously stated:

“Data were subjected to One-way ANOVA or Two-way ANOVA followed by Tukey's multiple comparison *post-hoc* test or Dunnett's multiple comparison post-hoc test using GraphPad Prism 5 software. Statistically significant differences between two mean values were established by a *p* < 0.05. Data are presented as the mean ± standard deviation.”

The corrected sentence appears below:

“*In vitro* data were subjected to One-way ANOVA or Two-way ANOVA followed by Tukey's multiple comparison *post-hoc* test or Dunnett's multiple comparison *post-hoc* test using GraphPad Prism 5 software. Data are presented as the mean ± standard deviation. *In vivo* results were compared using Mann-Whitney *U*-tests (for independent groups) or log-rank tests for survival data. These were conducted using R version 4.2.0. Statistically significant differences between two mean values were established by a *p* < 0.05.”

In the **Results** section there was an error in the section *Anti-inflammatory effect of Bt BEVs in vivo*, paragraph 2. This sentence previously stated:

“Mice orally administered with Bt BEVs exhibited a significantly higher (*p* < 0.01) survival rate throughout the experiment compared to mice that received vehicle (PBS) only (Figure 1B). Oral administration of Bt BEVs also contributed to a significant decrease (*p* < 0.05) in weight loss and disease activity index scores (Figures 1C, D).”

The corrected sentence appears below:

“Mice orally administered with Bt BEVs exhibited a higher but not significant survival rate throughout the experiment compared to mice that received vehicle (PBS) only (Figure 1B). Oral administration of Bt BEVs also contributed to a decrease in weight loss and disease activity index scores although this did not reach statistical significance (Figures 1C, D).”

In the **Discussion** section there was an error in the first and second paragraph. The first paragraph previously stated:

“In this study, we have provided *in vitro* and *in vivo* evidence for the anti-inflammatory and immunomodulatory properties of BEVs produced by the major human gut commensal bacterium Bt and identified the molecular basis of their interaction with monocytes and macrophages.”

The corrected sentence appears below:

“In this study, we have provided evidence for the anti-inflammatory and immunomodulatory properties of BEVs produced by the major human gut commensal bacterium Bt and identified the molecular basis of their interaction with monocytes and macrophages.”

The second paragraph of the **Discussion** previously stated:

“Oral administration of Bt BEVs ameliorates DSS-induced colitis in mice, underlining their potential as a treatment for non-infectious autoimmune pathologies.”

The corrected sentence appears below:

“From a small preliminary study, we found partial evidence that oral administration of Bt BEVs might ameliorate DSS-induced colitis in mice. However, the results were not statistically significant for most outcomes and so this requires confirmation in a properly powered study to establish their potential as a treatment for non-infectious autoimmune pathologies.”

In the **Conclusion** section there was an error. It was previously stated:

“Bt BEVs alleviated acute intestinal inflammation in DSS-treated mice, in association with increased IL-10 production.”

The corrected sentence appears below:

“*In vivo* studies provided some indication that Bt BEVs can improve survival, weight loss, disease activity and increased IL-10 production.”

The authors apologize for these errors and state that they do not change the scientific conclusions of the article in any way. The original article has been updated.

