# Supplementary figures and images for: Extracellular vesicles produced by the human gut commensal bacterium Bacteroides thetaiotaomicron elicit anti-inflammatory responses from innate immune cells
Source: Front Microbiol. 2022 Nov 10;13:1050271. doi: 10.3389/fmicb.2022.1050271 (PMC9684339; doi:10.3389/fmicb.2022.1050271)

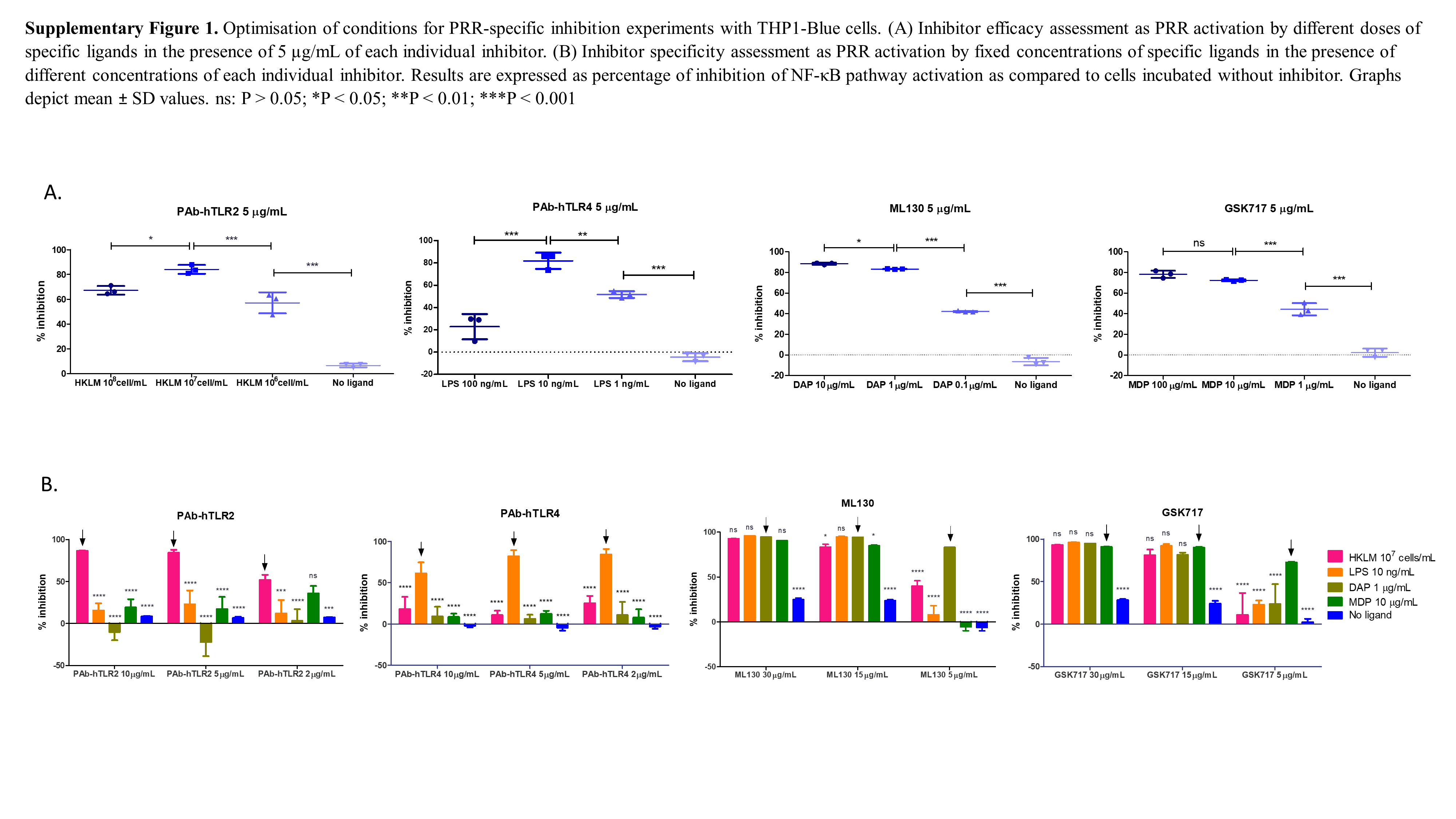

Supplement: Supplementary file 1 [file Image_1.TIF]
