# Supplementary material for: Extracellular vesicles produced by the human gut commensal bacterium Bacteroides thetaiotaomicron elicit anti-inflammatory responses from innate immune cells
Source: Front Microbiol. 2022 Nov 10;13:1050271. doi: 10.3389/fmicb.2022.1050271 (PMC9684339; doi:10.3389/fmicb.2022.1050271)
Supplement: Supplementary file 2 [file Table_1.DOCX]

**Supplementary Table 1.** Composition of Bacteroides Defined Medium (BDM4)

| **Compound** | **Concentration** |
| --- | --- |
| dH_2_O | N/A |
| KH_2_PO_4_ (for pH 7.4) | 30.4 mM |
| K_2_HPO_4_ ∙ 3H_2_O (for pH 7.4) | 69.6 mM |
| NaCl | 15 mM |
| (NH_4_)_2_SO_4_ | 8.5 mM |
| Protoporphyrin IX | 2 μM |
| Glucose | 30 mM |
| L-Histidine | 0.2 mM |
| L-Methionine | 200 uM |
| Vitamin K3 (Menadione) | 6 μM |
| MgCl_2_ | 0.1 mM |
| CaCl_2_ | 50 μM |
| FeSO_4_ ∙ 7H_2_O | 1.4 μM |
| L-Cysteine ∙ HCl | 4 mM |
