# Supplementary material for: Extracellular vesicles produced by the human gut commensal bacterium Bacteroides thetaiotaomicron elicit anti-inflammatory responses from innate immune cells
Source: Front Microbiol. 2022 Nov 10;13:1050271. doi: 10.3389/fmicb.2022.1050271 (PMC9684339; doi:10.3389/fmicb.2022.1050271)
Supplement: Supplementary file 3 [file Table_2.DOCX]

**Supplementary Table 2.** Disease Activity Index (DAI) scoring

| **Weight loss** | **Stool consistency** | **Bleeding** | **Appearance of caecum & colon** | **Appearance of caecum & colon contents** | **Score** |
| --- | --- | --- | --- | --- | --- |
| <1% | Well-formed pellets | None | Normal | Regular shape | 0 |
| 1-5% |  |  | White, abnormal size, strictures | Irregular but formed | 1 |
| 6-10% | Loose | Slight |  | Random shape | 2 |
| 11-15% |  |  |  | Blood in colon | 3 |
| >15% | Diarrhoea | Gross |  | Blood in caecum | 4 |
